# Supplementary material for: Spin-orbit torque in Pt/CoNiCo/Pt symmetric devices
Source: Sci Rep. 2016 Feb 9;6:20778. doi: 10.1038/srep20778 (PMC4746736; doi:10.1038/srep20778)
Supplement: Supplementary Information [file srep20778-s1.doc]

**Spin-orbit torque in Pt/CoNiCo/Pt symmetric devices**

Meiyin Yang^1†^, Kaiming Cai^1†^, Hailang Ju^2^, Kevin William Edmonds^3^, Guang Yang^4^, Shuai Liu^2^, Baohe Li^2^, Bao Zhang^1^, Yu Sheng^1^, ShouguoWang^4^, Yang Ji^1^, and Kaiyou Wang^1*^

*1. SKLSM, Institute of Semiconductors, CAS, P. O. Box 912, Beijing 100083, People’s Republic of China*

*2. Department of Physics, School of Sciences, Beijing Technology and Business University, Beijing 100048, China*

*3. School of Physics and Astronomy, University of Nottingham, Nottingham NG7 2RD, United Kingdom*

*4. State Key Laboratory of Magnetism, Institute of Physics, CAS, Beijing, 100190, China*

† These authors contributed equally to this work.

*Correspondence and requests for materials should be addressed to K. W. (e-mail: kywang@semi.ac.cn).

**S1. The magnetic properties of Pt/CoNiCo/Pt**

We investigated the magnetic anisotropy and damping of the symmetric Pt/CoNiCo/Pt by Ferromagnetic resonance (FMR). Fig. S1a shows the resonance field dependence of the angle between the external field and the film normal direction. The resonant field was wet at the frequency of 9 GHz. The resonance field increased with the external field tilting to the in-plane, indicating the perpendicular anisotropy. The curve could be fitted using the equation:

$\frac{2\pi f}{\gamma}=\sqrt{(Hr\cos(\theta_{H}-\theta_{M}))+H_{k}^{eff}\cos2\theta_{M})(Hr\cos(\theta_{H}-\theta_{M})+H_{k}^{eff}\cos^{2}\theta_{M})}$ (1)

where *f* is the frequency of the microwave, *γ* is the gyromagnetic ratio, *Hr* is the resonance field, *θ_H_* is the field to the film normal direction , *θ_M_* is the magnetization direction with the film normal and *H^k^_eff_* is the effective anisotropy field. The relationship of *θ_H_* and *θ_M_* follow the equation below:

$\sin2\theta_{M}=\frac{2Hr}{H_{k}^{eff}\sin(\theta_{H}-\theta_{M})}$ (2)

Using the equation (1) and (2), the curve in fig. S1b was perfectly fitted with the parameter of *γ* =1.1×10^11^ s^-1^T^-1^ and *H_k_^eff^* = 1600 Oe. The effective damping constant of the CoNi multilayer could be estimated with the equation:

$\Delta H\approx\frac{4\pi\alpha f}{\gamma}$ (3)

where *ΔH* is the half maximum of the field sweeping resonant curve in the Fig. S1c, *α* is the damping constant. The damping constant vs. field angle was calculated based on the equation (3). We use *α*=0.05 for the fitting curve in Fig. 2e when the field is in the perpendicular axis.


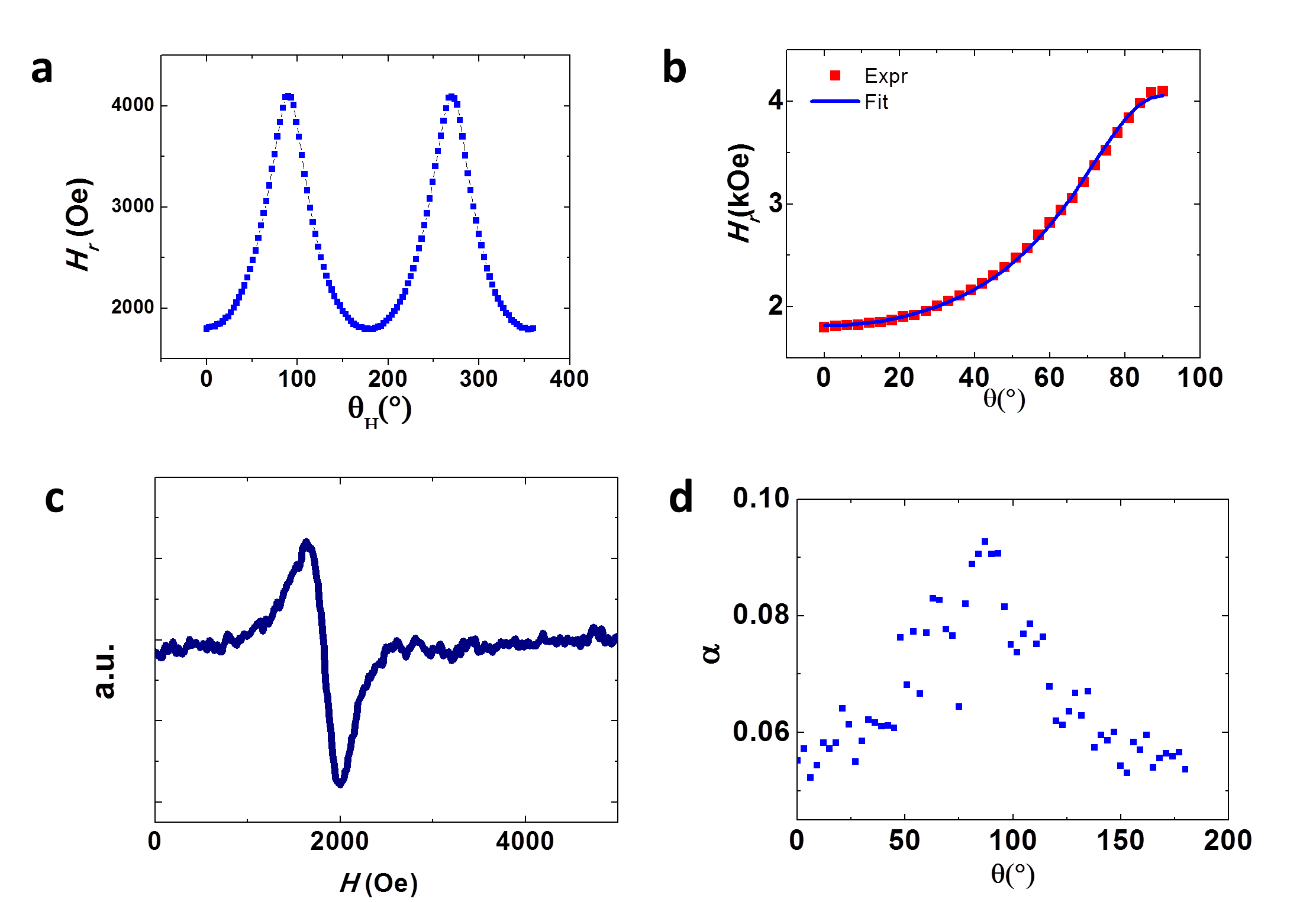


**Figure S1| FMR characterization of the Pt/CoNiCo/Pt thin film.** (**a**) Angular dependence of the resonance field. (**b**) The fitting of the curve of (a) from 0 to 90 degree. (**c**) The intensity of the resonance fields curve. (**d**) The relationship curve between the damping constant and the field applied angle.

**S2. Current distribution calculation**

To investigate the contribution of the spin Hall effect in the upper Pt, the current density distribution have to be calculated and mapped. The starting point for calculation of the current density distribution is Ohm’s law$\vec{j}=\sigma\vec{E}$, where *E* is the local electric field and conductivity is assumed to be a constant scalar here. The electrical field is the gradient field of the electrostatic potential *U*, so that $E=-\nabla U$. Charge conservation $\nabla E=0$ and thus$\nabla j=0$, which ultimately leads to $\nabla\left(\sigma\nabla U\right)=0$. If $\sigma$ is homogeneous, this equation converts into the elliptic differential equations which can be solved numerically with finite element method (FEM). Appropriate boundary conditions must be specified to obtain a unique solution. The boundary conditions are given by the fact that the current is not flowing perpendicular to the sample surface, thus, . For the leads, a known current density is entering and leaving the sample, the boundary condition is set as at the leads.

The resistivity of Co and Ni layers are quite similar ($\sigma_{\text{Co}}=62.4n\text{ }\Omega m$and $\sigma_{\text{Ni}}=69.3n\text{ }\Omega m$), so that we consider the CoNiCo layers as one layer with $\sigma_{\text{Pt}}=105n\text{ }\Omega m$, $\sigma_{CoNiCo}=62.4n\text{ }\Omega m$, $W=8\text{ }\mu m$,$h=2\text{ }nm$,$D=0.8\text{ }nm$and$H=3\text{ }nm$, the geometry shown in Fig. S2a. A total current *I*= 1 mA is flowing along the strip.

From the calculated results, cross section of charge current density distribution projection along the *x*-axis direction was shown in Fig. S1b. The current is mainly flowing through the strip in the bottom Pt layers. The average current density ratio is 1:1.5:17.5 for the top Pt, CoNiCo and bottom Pt layer.


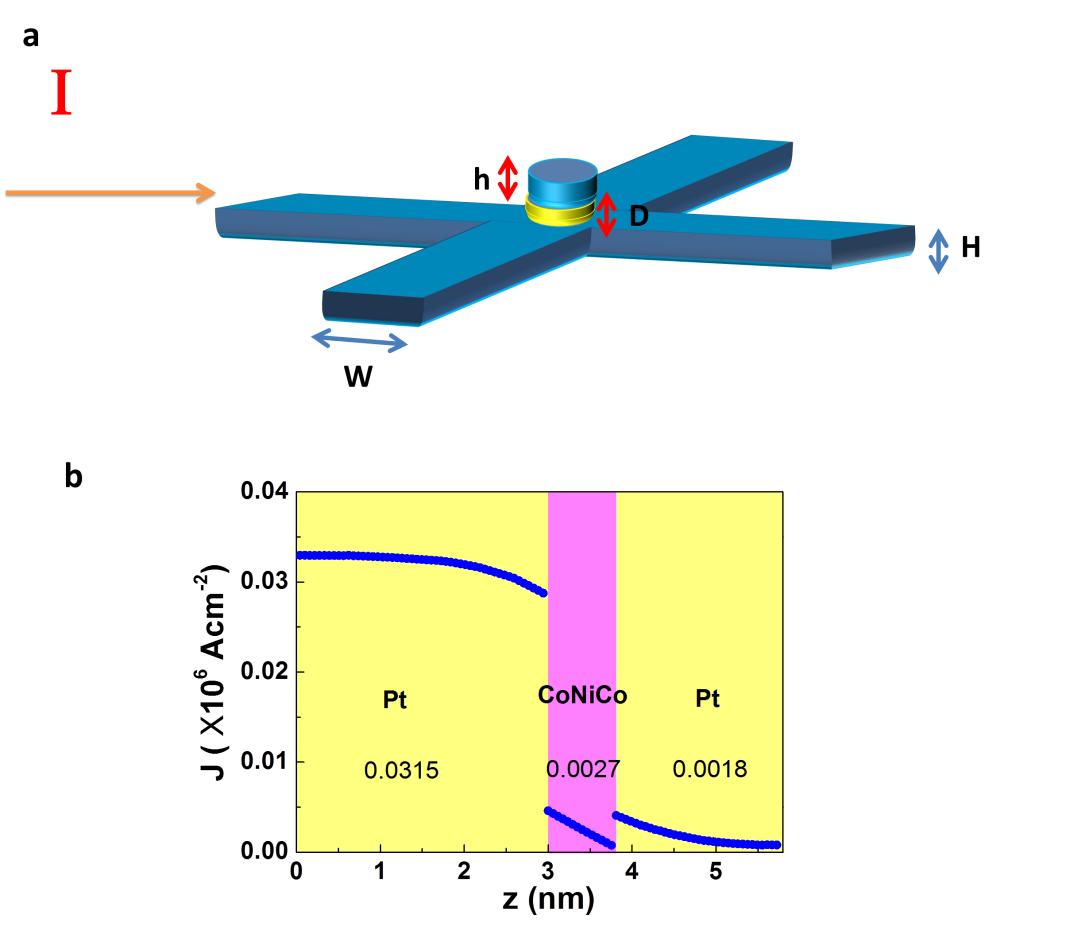


**Figure S2| Calculated charge current distribution in a Pt/CoNiCo/Pt device.** **(a**) The device geometry for the calculation. (**b**) In-plane current density projection current corresponding to the device depth. Current is mainly flowing through the Pt layer. We merge the Co layers and Ni layer as one mixed layer, for the conductivity of Co layer is approximately equal to that of Ni layer. We use the $\sigma_{Pt}/\sigma_{CoNiCo}=1.6$. When the total current is 1mA, the current density of bottom Pt layer is about $3.3\times10^{6}\text{ Acm}^{\text{-2}}$.

**S3. Effect of the Oersted field**

The current flowing through the Pt layer can generate Oersted field which can also act on the CoNiCo magnetization as a transverse effective field. The direction of the Oersted field coincides with *H*_T_ here. In order to estimate its effect, we estimated the magnitude of the Oersted field according to the current distribution at a fixed current 1mA.


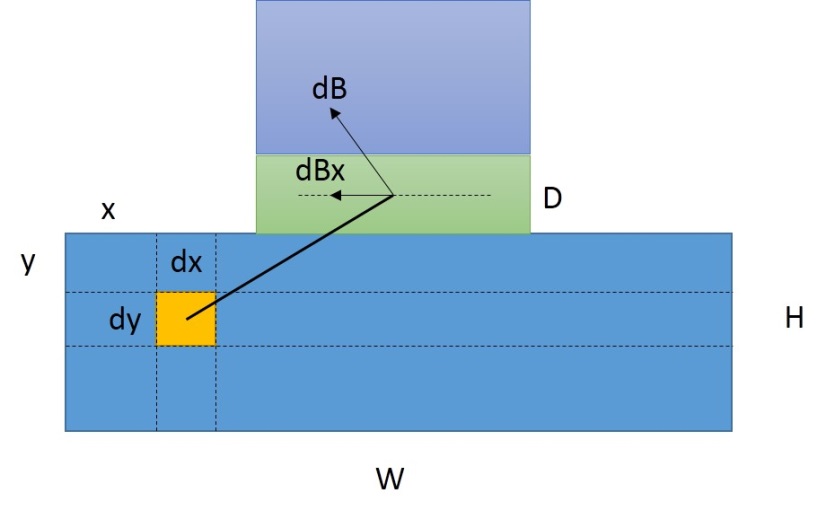


**Figure S3| Schematic cross section of the device.** The bottom Pt layer with width W and thick is H. D is thickness of the CoNiCo.

From the current distribution, the current is mostly flowing through the bottom Pt strip. To get an analytic solution, we can estimate the current density as $J=I/\left(WH\right)$, where $W$and $H$ are defined in Fig. S3. This equation assumes that the current flows uniformly in the Pt layer and nearly no current flows through CoNiCo. This is a reasonable approximation from the current distribution calculation results.

According to the Biot-Savart law, the magnetic field *B* is calculated by $dB=\frac{\mu_{0}}{2\pi}\frac{dI}{r_{0}}$. We get to know that the magnetic field produced by the current dI on the *x*-axis direction as:

$dB_{x}=\frac{\mu_{0}}{2\pi}\frac{dI}{r_{0}}\sin\theta=\frac{\mu_{0}}{2\pi}\frac{Jdxdy}{\sqrt{\left(\frac{W}{2}-x\right)^{2}+\left(\frac{D}{2}+y\right)^{2}}}\frac{\left(\frac{D}{2}+y\right)}{\sqrt{\left(\frac{W}{2}-x\right)^{2}+\left(\frac{D}{2}+y\right)^{2}}}$ (4)

Then, the total magnetic field can be written as:

$B=\int_{y=0}^{H} \int_{x=0}^{W} dBx=\frac{\mu_{0}J}{2\pi}\int_{y=0}^{H} \int_{x=0}^{W} \frac{\left(\frac{D}{2}+y\right)}{\left(\frac{W}{2}-x\right)^{2}+\left(\frac{D}{2}+y\right)^{2}}dxdy$ (5)

For $W\gg H\gg y$，$W\gg D$，

$I_{1}=\int_{x=0}^{W} \frac{\left(\frac{D}{2}+y\right)}{\left(\frac{W}{2}-x\right)^{2}+\left(\frac{D}{2}+y\right)^{2}}dx=2\arctan\left(\frac{\frac{W}{2}}{\frac{D}{2}+y}\right)\approx\pi$ (6)

Then, substitute in the previous equation,

$B_{Oersted}=\int_{y=0}^{H} \int_{x=0}^{W} dBx=\frac{\mu_{0}J}{2\pi}\int_{y=0}^{H} \pi dy=\frac{\mu_{0}J}{2\pi}\left(\int_{y=0}^{H} \pi dy\right)=\frac{\mu_{0}JH}{2}$ (7)

If change the current density in the current,

$B=\frac{\mu_{0}JH}{2}=\frac{\mu_{0}JWH}{2W}=\frac{\mu_{0}I}{2W}$ (8)

With current *I*=1mA，the Oersted field is determined to be 0.6 Gs.

$$B=\frac{\mu_{0}JH}{2}=\frac{\mu_{0}JWH}{2W}=\frac{\mu_{0}I}{2W}=\frac{4\pi\times10^{-7}\times1\times10^{-3}}{2\times10\times10^{-6}}=2\pi\times10^{-5}\text{ T}=0.628\text{ Gs}$$

Since the resulting Oersted field from the electrical current is only 0.6 Oe, it is only 6% compared with the *H_DL_*. Thus, the effect of the Oersted field is negligible in our analysis.

**S4. Planar Hall effect**

The R_PHE_ is measured by applying an in-plane field at 7000 Oe to saturate the M and rotating the field in the plane and recording its Hall voltage, which could be found in Fig. S4.


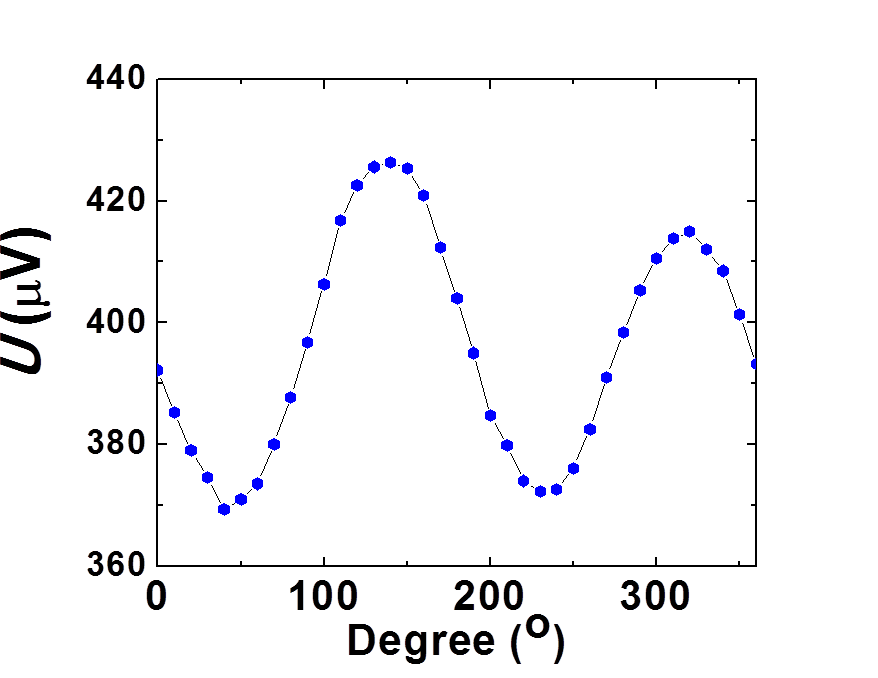


**Figure S4| Planar Hall effect of the device.**

**S5. The harmonic measurements of Pt/CoNiCo/Pt Hall bar**

We patterned the thin film only into a Hall bar with 20 μm wide and performed the harmonic measurements. In this case, the current is flowing in both upper and lower Pt layer. We did not observed any second harmonic signal when applying the magnetic field along *y* axis. The damping-like effective field from measurements is ~8 Oe per 10^7^ Acm^-2^ which is only less than 30% compared with the patterned sample (~25 Oe per 10^7^ Acm^-2^), indicating the spin-orbit torque from lower and upper Pt cancelled with each other. The results also proved the enhancement of the spin-orbit torque from the device design.


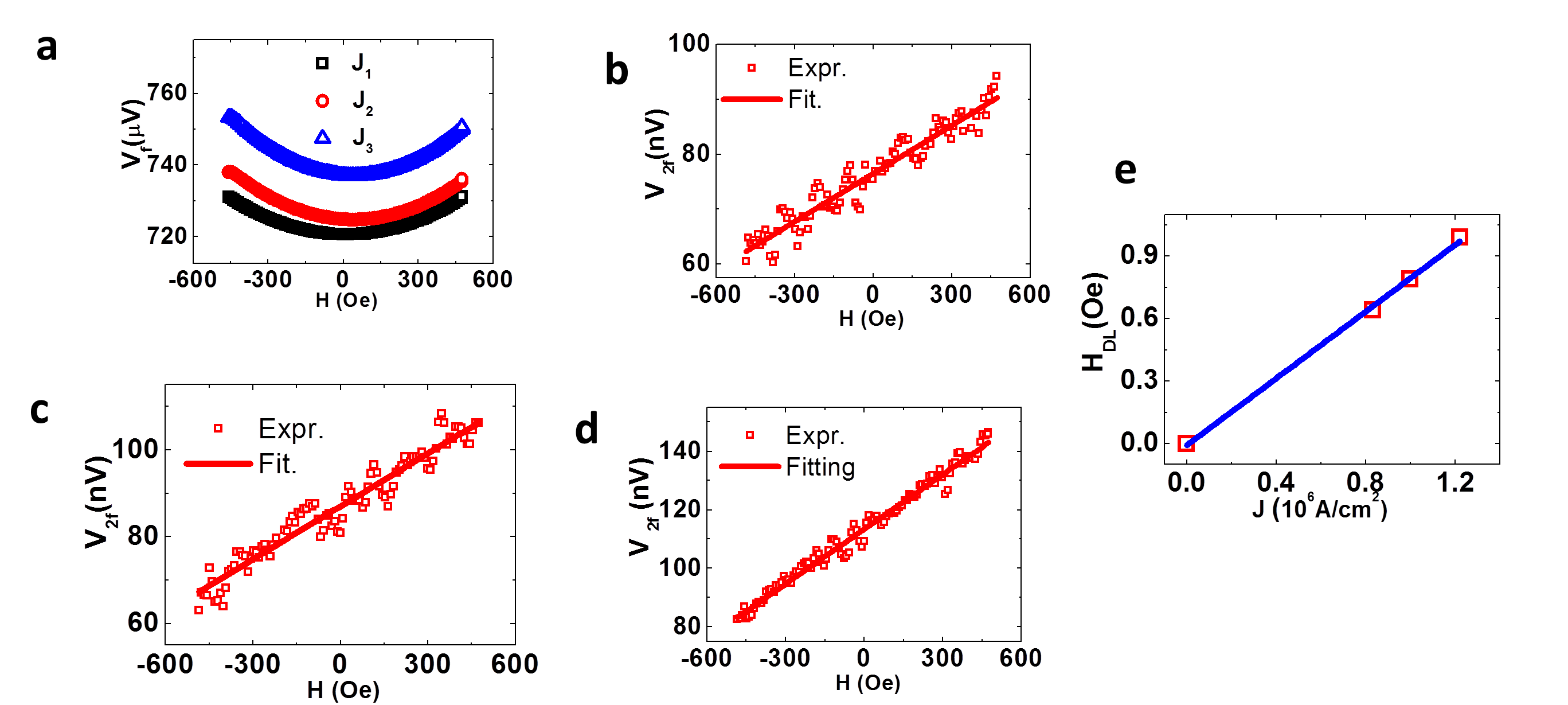


**Figure S5| The harmonic measurements of the unpatterned sample .**(a) The first harmonic voltage signal. The second harmonic voltage of (b)*J_1_*= 8.3×10^5^ Acm^-2^, (b)*J_2_*= 1×10^6^ Acm^-2^, (c) *J_3_*=1.2×10^6^ Acm^-2^ ,and (d) the damping-like effective field as a function of the current density.

**S6. Current switching under in-plane magnetic fields**

The deterministic switching could be realized by applying an in-plane magnetic field along *x* axis. To investigate the magnetic fields dependence of the fields angle with the current *θ*, the current switching loop under field of 400 Oe with different direction in-the plane were conducted as is shown in Fig. S6(a). With the fields titling from 0 (along x axis) to 90 degree (along *y* axis), the loop became smaller, indicating the deterministic switching gradually vanished. The switching current densities are equal with both positive and negative current, suggesting the in-plane field direction did not have a noticeable influence on the critical switching density. The *R_h_* decreased with increasing the angle obtained from the Fig. S6a as is plotted in Fig. S6b. The loosing of *R_h_* is due to the decreasing field projection along x axis when rotating the magnetic field from 0 to 90 degree. Fig. S6c simulated the switching dynamics of *M_z_* under 200 Oe in-plane magnetic field during the current pulse of 5 ns with field -like torque (*β*=1) and no field-like torque (*β*=0) by LLG micromagnetic simulator. No significant difference concerning about the switching of *M_z_* are found, indicating that the *H_FL_* (or external fields along *y* axis) did not contribute much to the deterministic switching, which is consistent with the experiment results.


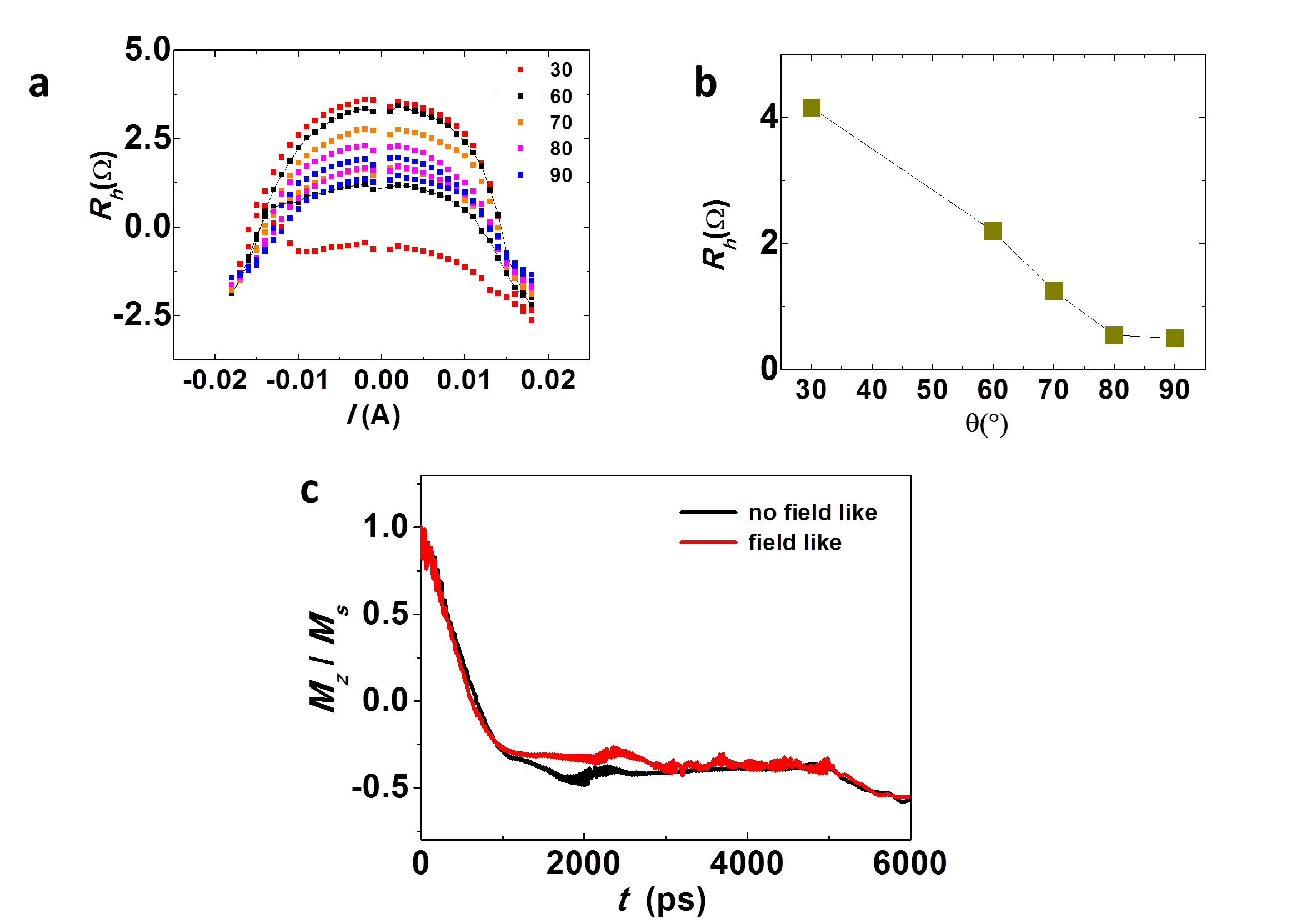


**Figure S6| In-plane magnetic fields acting on the current switching loop.** (**a**) The switching loop with the external fields applied from different angle. (**b**) The *R_h_* variation as a results of the current switching under different fields direction applied. (**c**) The averaged *M_z_*/*M_s_* during the current pulse.

**S7. Domain dynamics during the switching**

The *M* projections along *x*, *y* and *z* axis could be also obtained during the current-induced magnetization switching under an in-plane field by the micromagnetic simulation (Fig. S7). The red color represented the positive sign of the magnetization and blue indicated the negative one. During the current pulse (5 ns), two domains with the domain walls containing most *–y* magnetization were stabilized. Although, the external fields were applied along the *+x* axis, the domain with *+x* magnetization did not grow into a majority domain during the current switching. The domains geometry was balanced by the dipole interaction, the *Hx* external field and the spin-orbital torque. After the current pulse (6.6 ns), the domain wall formed +*x* magnetization due to the *H_x_* external field.


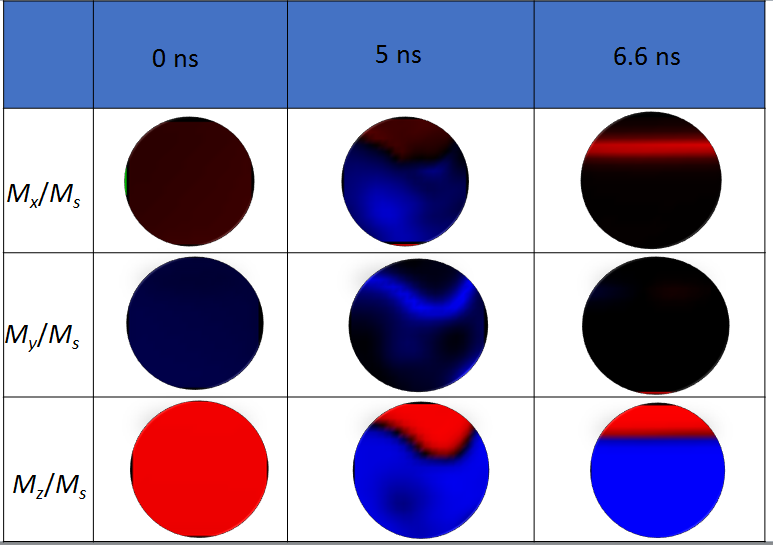


**Figure S7| The simulated domain structure under in-plane field at different time.** The *M* is projected along *x, y* and *z* axis at different time by the micromagnetic simulation.

Figure S8 compared the *M_z_/M_s_* mapping between the 100 Oe and 400 Oe external fields along *+x* axis during the current pulse and after the pulse. During the pulse, the –*M_z_* domain under 400 Oe field grew larger than the one with 100 Oe *H_x_* field. After the current pulse, the domain wall continued moved to enlarge the –*M_z_* domain until it covered the entire magnet.


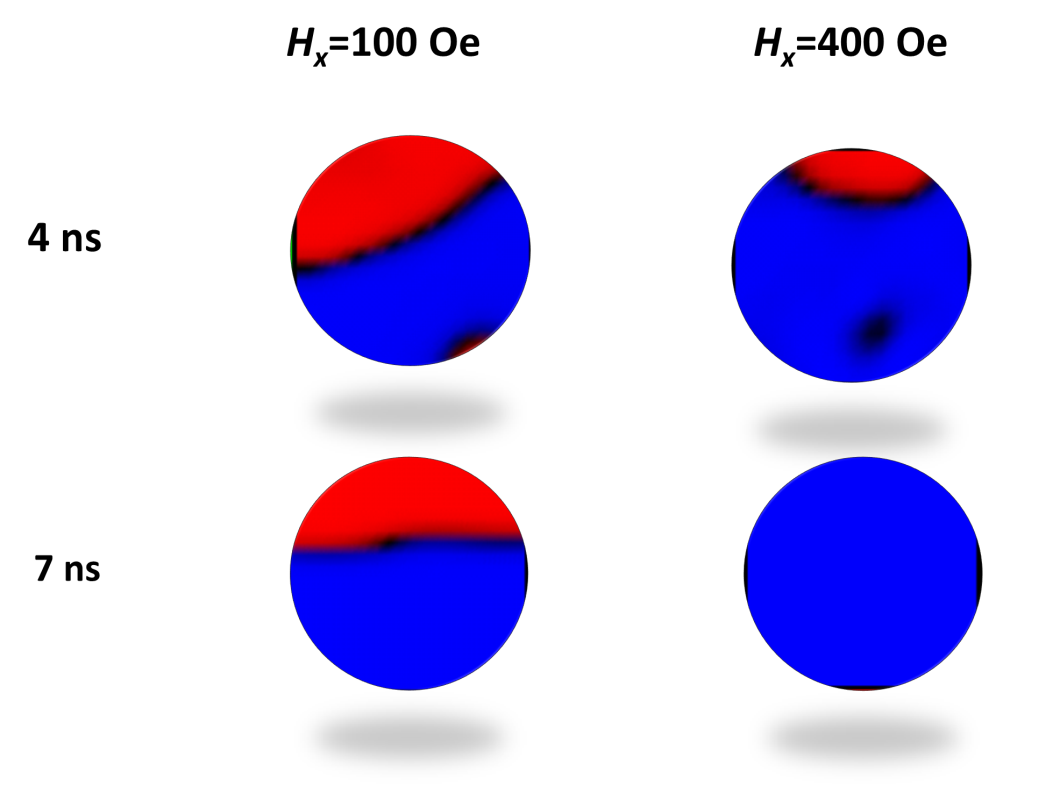


**Figure S8| Domains of *M_z_* during the current pulse and after the current pulse for *H_x_*=100 Oe and *H_x_*=400 Oe.**
